# Investigation of Cytotoxicity and Cell Uptake of Cationic Beta-Cyclodextrins as Valid Tools in Nasal Delivery

**DOI:** 10.3390/pharmaceutics12070658

**Published:** 2020-07-12

**Authors:** Giovanna Rassu, Silvia Fancello, Marta Roldo, Milo Malanga, Lajos Szente, Rossana Migheli, Elisabetta Gavini, Paolo Giunchedi

**Affiliations:** 1 Department of Chemistry and Pharmacy, University of Sassari, via Muroni 23/a, 07100 Sassari, Italy; grassu@uniss.it (G.R.); pgiunc@uniss.it (P.G.); 2 Department of Medical, Surgical and Experimental Sciences, University of Sassari, viale San Pietro 43/b, 07100 Sassari, Italy; sfancello@uniss.it; 3 School of Pharmacy and Biomedical Sciences, University of Portsmouth, St Michael’s Building, White Swan Road, Portsmouth PO1 2DT, UK; marta.roldo@port.ac.uk; 4 CycloLab Ltd., Illatos út 7, H-1097 Budapest, Hungary; malanga@cyclolab.hu (M.M.); szente@cyclolab.hu (L.S.)

**Keywords:** cationic cyclodextrin, cyclodextrin polymer, epichlorohydrin cross-linker, nasal delivery, cytotoxicity, cell uptake

## Abstract

Cyclodextrin polymers have high applicability in pharmaceutical formulations due to better biocompatibility, solubility enhancement, loading capacity and controlled drug release than their parent, cyclodextrins. The cytotoxicity and cell uptake of new cationic beta-cyclodextrin monomers and polymers were evaluated as suitable materials for nasal formulations and their protective effects on cells exposed to hydrogen peroxide were studied. PC12 and CACO-2 cells were selected as the neuronal- and epithelial-type cells, respectively, to mimic the structure of respiratory and olfactory epithelia of the nasal cavity. All cationic beta-cyclodextrin polymers tested showed dose- and time-dependent toxicity; nevertheless, at 5 µM concentration and 60 min of exposure, the quaternary-ammonium-beta-cyclodextrin soluble polymer could be recognized as nontoxic. Based on these results, a fluorescently labelled quaternary-ammonium-beta-cyclodextrin monomer and polymer were selected for uptake studies in CACO-2 cells. The monomeric and polymeric beta-cyclodextrins were internalized in the cytoplasm of CACO-2 cells; the cationic monomer showed higher permeability than the hydroxypropyl-beta-cyclodextrin, employed as comparison. Therefore, these cationic beta-cyclodextrins showed potential as excipients able to improve the nasal absorption of drugs. Furthermore, amino-beta-cyclodextrin and beta-cyclodextrin soluble polymers were able to reduce oxidative damage in PC12 and CACO-2 cells and thus could be studied as bioactive carriers or potential drugs for cell protection against oxidative stress.

## 1. Introduction

Cyclodextrins (CDs) are very interesting excipients widely used in the pharmaceutical field due to their peculiar properties. CDs are cyclic oligosaccharides, formed of α-D-glucopyranose units, with a characteristic truncated cone shape having a hydrophilic outer surface and hydrophobic interior cavity [1]. Therefore, CDs are able to entrap hydrophobic drugs in their cavities forming an inclusion complex, which favours drug dissolution in the aqueous phase, protects the drug from chemical and enzymatic degradation and limits drug toxic effects [1,2]. Moreover, CDs act as penetration enhancers and this property, in addition to the increased water solubility, leads to an improvement of the bioavailability of drugs. The ability to enhance drug crossing through biological barriers is in part due to interactions of CDs with lipid components of the membrane that induce a perturbation in the fluidity and permeability [3].

Starting from native CDs (named α-CD, β-CD and γ-CD), more than 1500 CD derivatives have been synthetized in order to improve their solubility and minimize toxicity [4]. Among the class of β-cyclodextrin derivatives, (2-hydroxypropyl) beta-cyclodextrin (HP) is an approved pharmaceutical excipient and its monograph has been published in both the European Pharmacopoeia and United States Pharmacopoeia [5]. HP is recognized also as an active pharmaceutical ingredient and received the orphan drug designation for the treatment of Niemann–Pick disease from the US Food and Drug Administration (FDA) and the European Medicines Agency (EMA) [5,6,7,8]. Moreover, HP is able to provide protection against neurotoxicity induced by β-amyloid, and therefore useful in the treatment of Alzheimer’s disease [8,9,10]. HP and other CD derivatives showed a potential therapeutic use in neurodegenerative diseases, stroke, neuro-infections and brain tumors [4,11].

CDs and their derivatives have also been polycondensed with a cross-linking agent, such as epichlorohydrin, to obtain polymeric networks with better biocompatibility and solubility enhancement than parent CDs as well as superior loading capacity and controlled drug release [12,13]. Cyclodextrin polymers (CDPs) are high-molecular-weight compounds, they can be either soluble or insoluble in water, and be found as positively or negatively charged or nonionic in nature [13]. Charged soluble CDPs possess special complexing and solubilizing ability due to ionic interactions with ionic drugs [13] such as insulin [14], DNA [15] and siRNA [16] and, thus, have been proposed as drug or gene carrier systems. Nevertheless, CDPs have been studied only for oral and topical delivery [13].

On the basis of these assumptions, the aims of this work were to evaluate the cytotoxicity and biocompatibility of cationic beta-cyclodextrin monomers and polymers and to explore their applicability in nasal formulations. The positively charged beta-cyclodextrins could be advantageously used to increase the residence time of formulations in the nasal cavity due to the ionic interactions with the negative charges of mucus [17]. The influence of molecular weight and charge of CDPs on cell viability was also investigated. In particular, the in vitro toxicity of cationic CDPs was evaluated and compared to that of parent cyclodextrin and HPCD, selected as standard. CACO-2 and PC12 cells were chosen as model of epithelial and neuronlike phenotypes, respectively to predict the effect of the cationic beta-cyclodextrins in respiratory and olfactory mucosa of the nasal cavity [18]. By using fluorescently labelled beta-cyclodextrins, uptake and permeation studies were performed.

Finally, in order to find a potential therapeutic activity of these compounds, the protective effects against oxidative stress on cells exposed to hydrogen peroxide were evaluated.

## 2. Materials and Methods

### 2.1. Materials

Dulbecco’s modified Eagle’s medium (DMEM/F12, HEPES, no phenol red), fetal bovine serum (FBS), horse serum (HS), streptomycin/penicillin, nonessential amino-acids, L-glutamine and trypsin were purchased from Life Technologies Italia (Monza, Italy). Hydrogen peroxide (H_2_O_2_, 30%), 3-(4,5-Dimethyl-thiazol-2-yl)-2,5,diphenyltetrazoliumbromide (MTT, 97.5%), rhodamine B isothiocyanate, paraformaldehyde solution (adjusted to pH 7.4) solution, Hank’s salt (HBSS) solution, 3-(4,5-Dimethyl-thiazol-2-yl)-2,5,diphenyltetrazoliumbromide triton X-100 were acquired from Sigma-Aldrich (Milan, Italy) Beta-cyclodextrins (fluorescent or not) were from CycloLab Ltd. (Budapest, Hungary).

### 2.2. Beta-Cyclodextrins

Beta-cyclodextrin derivatives were produced and characterized by CycloLab as follows—(2-hydroxy-3-N,N,N-trimethylamino)propyl-beta-cyclodextrin chloride (QA), quaternary- ammonium-beta-cyclodextrin soluble polymer crosslinked with epichlorohydrin (QAPS) and quaternary ammonium-6-deoxy-6-((5/6)-rhodaminylthioureido)- (2-Hydroxy-3-N,N,N-trimethylamino)-beta-cyclodextrin (RBITC-QA) were obtained by solubilizing in alkaline conditions native beta-cyclodextrin, beta-cyclodextrin soluble polymer and 6-deoxy-6-((5/6)-rhodaminylthioureido)-beta-cyclodextrin, respectively, and by alkylating the reaction mixtures with glycidyltrimethylammonium chloride for 12 h at room temperature. Extensive dialysis by using a low cut-off (100–500 Da) dialysis membrane and freeze drying yielded the corresponding positively charged quaternary ammonium CD compounds. Quaternary-ammonium-rhodamine-labelled beta-cyclodextrin soluble polymer crosslinked with epichlorohydrin (RBITC-QAPS) was synthesized as previously reported [19]. Heptakis (6-deoxy-6-amino)-beta-cyclodextrin heptahydrochloride (HA) was prepared according to a previously described procedure [20]. Beta-cyclodextrin soluble polymer crosslinked with epichlorohydrin (PS), amino-beta-cyclodextrin soluble polymer crosslinked with epichlorohydrin (HAPS), rhodamine labelled beta-cyclodextrin soluble polymer crosslinked with epichlorohydrin (RBITC-PS) were prepared according to synthetic procedures previously described [21]. 2-Hydroxypropyl-beta-cyclodextrin (HP) and 6-deoxy-6-((5/6)-rhodaminylthioureido)-hydroxypropyl-beta-cyclodextrin (RBITC-HP) can be prepared according to the synthetic descriptions previously reported [22].

The main properties of CDs utilized in the study are reported in Table 1; further information of analogue fluorescent cyclodextrins are shown in Table S1 (Supplementary Materials).

In Figures S1 and S2 (Supplementary Materials), cartoon representations for the CD monomers and polymers investigated in the study are shown.

### 2.3. Cell Culture and Cyclodextrins (CDs) Treatments

Rat pheochromocytoma-derived cell line (ATCCCRL-1721) PC12 cells (passage 12–25) were cultured in atmosphere of 5% CO_2_/95% humidified air at 37 °C in 60 mm plastic culture plates in Dulbecco’s modified Eagle’s medium (DMEM/F12) with 10% Horse Serum (HS), 5% Fetal Bovine Serum (FBS) and 1% of penicillin/streptomycin. CACO-2 cells (European Collection of Cell Cultures (ECACC UK) (passage 20–40) were maintained in tissue culture flask T75 in Dulbecco’s modified Eagle’s medium (DMEM) with 10% FBS, 1% nonessential amino acids, 1% L-glutamine and 1% penicillin–streptomycin solution and maintained at 37 °C and 5% CO_2_. The experiments were performed in the cell lines exposed to the different beta-cyclodextrin monomers and polymers (HP, QA, QAPS, HA, HAPS and PS) at different concentrations and times of exposure.

### 2.4. In Vitro Cell Viability Studies

Caco-2 and PC12 cells were used as model of epithelial and neuronlike phenotypes, respectively [16]. PC12 and CACO-2 cells were treated with the different concentrations of beta-cyclodextrin monomers and polymers (0.5‒1‒2.5‒5‒10 µM) for 15, 30, 60 min and 24 h. At the end of the experiments, the cell viability was assessed by the 3-(4,5-dimethyl-thiazol-2-yl)-2,5, diphenyltetrazolium bromide (MTT) assay. Viable cells convert the soluble dye MTT to insoluble formazan crystals. One mg/mL of MTT was added for each sample and incubated for 4 h at 37 °C. MTT was removed, the cells rinsed with PBS and centrifuged, the supernatant discarded and the pellet was dissolved in 2 mL of isopropanol. The absorbance values were detected at 578 nm (Diagnostic Microplate Reader, BioTek UK, Swindon, United Kingdom). For the experiments, 1 × 10^5^ cells/mL/well were seeded in 24-well plates, performing all experiments in triplicate. The result was expressed as cell viability in a percentage according to the following formula:

(1) $$\mathrm{Cell}\;\mathrm{viability}\;\left(\%\right)=\frac{{\mathrm{OD}}_{\mathrm{treated}\;\mathrm{cells}}}{{\mathrm{OD}}_{\mathrm{untreated}\;\mathrm{cells}}}\times 100$$

where ${\mathrm{OD}}_{\mathrm{treated}\;\mathrm{cells}}$ was the optical density of cells treated with the beta-cyclodextrin monomers and polymers and ${\mathrm{OD}}_{\mathrm{untreated}\;\mathrm{cells}}$ was the optical density of untreated cells used as control.

### 2.5. Uptake and Permeation Studies

#### 2.5.1. Confocal Microscopy

CACO-2 cells (seeded on coverslip glass at the density 200,000 cells/mL/well) treated with RBITC-HA, RBITC-PS and RBITC-QAPS (5 µM) for 30 min were analysed by confocal microscopy. After the treatment, the cells were washed several times with PBS in order to eliminate the labelled beta-cyclodextrins located outside the cells; then, CACO-2 cells were fixed with 4% paraformaldehyde solution. The nuclei were stained with DAPI 2 µg/mL in PBS for 15 min and the coverslips were sealed on microscope slides. Samples were observed by a confocal laser-scanning (Rhodamine B isothiocyanate excitation: 568 nm HeNe laser; emission: 623 nm) microscope equipped with a Plan Apochromatic 63x DIC oil objective (NA1.4) (LSM710; Zeiss, Oberkochen, Germany). The images were analysed with the software Zen2008 Light Edition (Zeiss, Oberkochen, Germany).

#### 2.5.2. Relative Beta-Cyclodextrins Uptake

CACO-2 cells were seeded in 96-well black plate at the density of 25,000 cells/well and exposed to RBITC-HA, RBITC-PS and RBITC.QAPS (5 µM) dissolved in HBSS for 15, 30 and 60 min. At the end of the incubation, the cells were washed 4 times with HBSS and the cell lysis was performed with 1% Triton X-100. The fluorescence intensity was measured by the microplate reader SpectraMax i3x (Molecular Devices, LLC. San Jose, CA, USA) (rhodamine B isothiocyanate excitation: 568 nm and emission: 623 nm).

#### 2.5.3. Uptake and Transepithelial Permeability Evaluation in Cell Monolayers

CACO-2 cells were seeded at the density of 200,000 cells/0.5 mL in cell culture inserts for a 12-well plate pretreated with collagen (pore size 0.4 µm, growth area 1.12 cm^2^) and the basolateral compartments were filled with 1.5 mL of complete medium. The cells were cultured for 21 days to obtain differentiated cell monolayers and the medium was replaced twice per week. The monolayer integrity was evaluated measuring the transepithelial electrical resistance (TEER, expressed in Ω cm^−2^) (EVOM Meter, World Precision Instruments, Germany GmbH, Friedberg, Germany) CACO-2 cell monolayers were used for the experiments when TEER values were higher than 700 Ω cm^−2^. Before treatments, the monolayers were washed twice with PBS, preincubated in HBSS for 20 min and then exposed apically to the RBITC-HA, RBITC-PS and RBITC-QAPS (5 µM) in HBSS for 15, 30 and 60 min at 37 °C. Samples derived from the basolateral compartments were collected for each exposure time and the volumes replenished with HBSS. The monolayers were washed four times with HBSS and lysed with 1% Triton-X100. The fluorescence intensity in the intracellular and in the corresponding basolateral samples was measured by the microplate reader SpectraMax i3x (Molecular Devices, LLC., San Jose, CA, USA) (Rhodamine B isothiocyanate excitation: 568 nm and emission: 623 nm). The obtained values were used to calculate the apical to basolateral permeation rate across the monolayers measuring the apparent permeability coefficients:

P_app_ = (dQ/dt) × (1/C_0_ × A) (2)

where P_app_ is the apparent permeability coefficient (cm/s), dQ/dt is the permeability rate of beta-cyclodextrins (mol/s), C_0_ is the initial concentration of beta-cyclodextrins in the apical chamber (mol/mL) and A is the surface area of the membrane (cm^2^).

### 2.6. Studies of Protective Effects

PC12 and CACO-2 cells were exposed to hydrogen peroxide (75 μM) in the presence of HP, QA, QAPS, HA, NH_2_-PS and PS (0.5 µM) for 24 h. At the end of the experiments, the cell viability was assessed by the 3-(4,5-dimethyl- thiazol-2-yl)-2,5, diphenyltetrazolium bromide (MTT) assay. Viable cells convert the soluble dye MTT to insoluble formazan crystals. A total of 1 mg/mL of MTT was added for each sample and incubated for 4 h at 37 °C. MTT was removed, the cells rinsed with PBS and centrifuged, the supernatant discarded and the pellet was dissolved in 2 mL of isopropanol. The absorbance values were detected at 578 nm (Diagnostic Microplate Reader, BioTek UK, Swindon, United Kingdom). For the experiments, 1 × 10^5^ cells/mL/well were seeded in 24-well plates, performing all experiments in triplicate.

### 2.7. Statistical Analysis

All experiments were reported as mean values with 95% confidence intervals. Statistical significance (control vs. experimental groups) was evaluated by One-Way ANOVA analysis of variance test using Graph-Pad Prism 5.0 software (GraphPad Software, Inc, San Diego, CA, USA).

## 3. Results

### 3.1. In Vitro Cell Viability Studies

**Figure 1:**
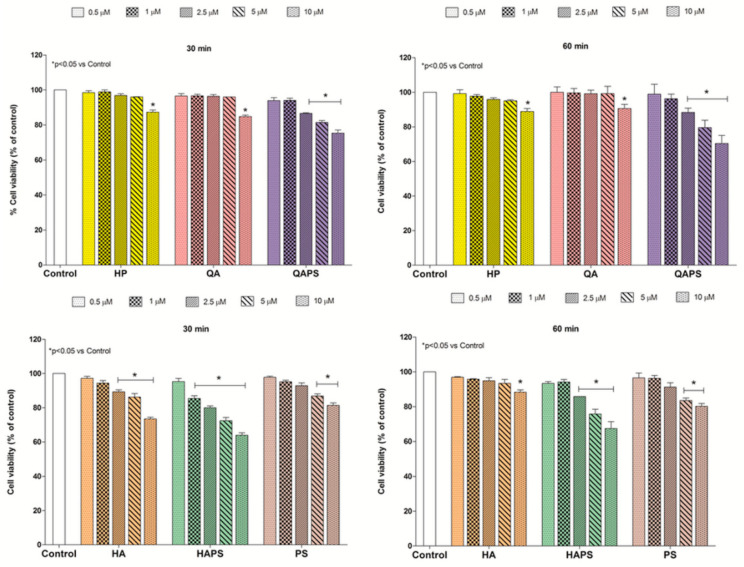
Effect of different concentrations (0.5–1–2.5–5–10 µM) of HP, QA, QAPS, HA, HAPS and PS on PC12 cell viability at increasing times of exposure (30 and 60 min). Data are reported as mean ± SD (*n* = 3). * *p* < 0.05 vs. Control (ANOVA test).

**Figure 2:**
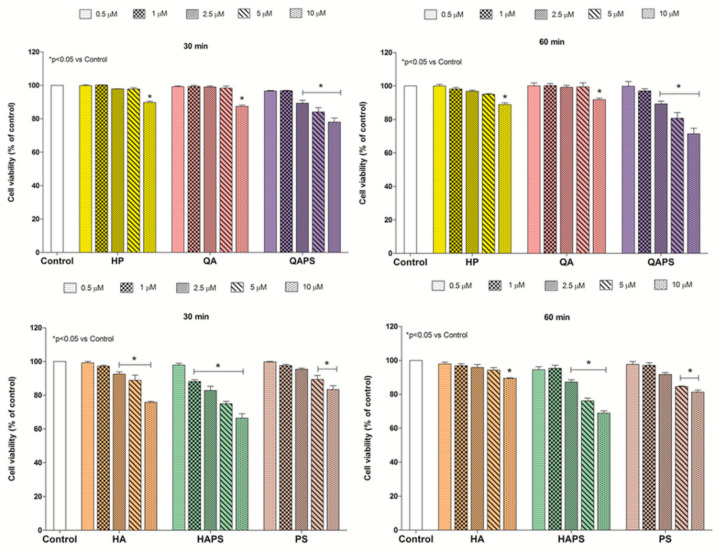
Effect of different concentrations (0.5–1–2.5–5–10 µM) of HP, QA, QAPS, HA, HAPS and PS on CACO-2 cell viability at increasing times of exposure (30 and 60 min). Data are reported as mean ± SD (*n* = 3). * *p* < 0.05 vs. Control (ANOVA test).

Figure 1 and Figure 2 show the effects of HP, QA, QAPS, HA, HAPS and PS at different concentrations and exposure times (30 and 60 min) on PC12 and CACO-2 cell viability, respectively. Almost all beta-cyclodextrins tested at 30 and 60 min did not induced a significant decrease of cell viability until the concentration of 5 µM in both cell lines without differences between the two time points. Only QAPS and HAPS showed a slight decrease in cell viability at 30 and 60 min of treatment at the highest concentration of 10 µM. HP, QA and QAPS did not show toxic effects on CACO-2 cells up to 5 µM concentration (Figure 2). On the basis of these data, the concentration of 5 µM of HP, QA and QAPS was used in the following studies in CACO-2 cells.

Other experiments were performed at higher exposure times (24 h) as shown in Figure S3–S6 of the Supplementary Material. In particular, at 24 h of exposure, QAPS showed lower toxicity in PC12 cells than CACO-2 cells at all concentrations.

### 3.2. Uptake Studies by Confocal Microscopy

**Figure 3:**
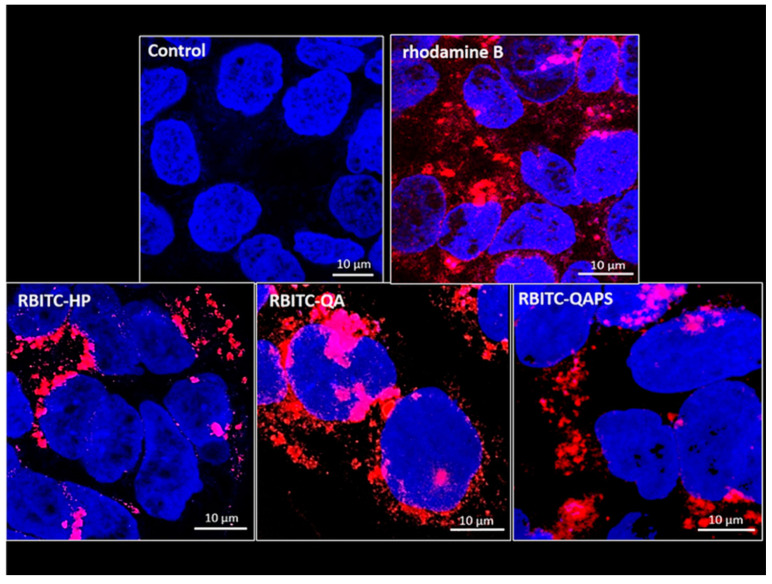
Uptake analysis of the fluorescence of RBITC-HP, RBITC-QA and RBITC-QAPS (5 µM) in CACO-2 cells by confocal microscopy after 30 min of exposure. The figure shows the internalization by CACO-2 cells of the different labelled beta-cyclodextrins in comparison with untreated cells or treated with rhodamine not conjugated (blue channel—nuclear stain; red channel—rhodamine alone or conjugated with beta-cyclodextrins).

The uptake study was performed using the fluorescent rhodamine-conjugated RBITC-HP, RBITC-QA and RBITC-QAPS (5 µM, chosen on the basis of the cell viability data) to demonstrate their internalization in CACO-2 cells after 30 min from treatments. The confocal microscopy analysis (Figure 3) showed that all labelled beta-cyclodextrins tested (red) were able to enter in CACO-2 cells, placing in close proximity to the nucleus (blue), but with different efficiency. In particular, the uptake of the monomer RBITC-QA was higher than RBITC-HP and the polymer RBITC-QAPS.

### 3.3. Relative Beta-Cyclodextrins Uptake

**Figure 4:**
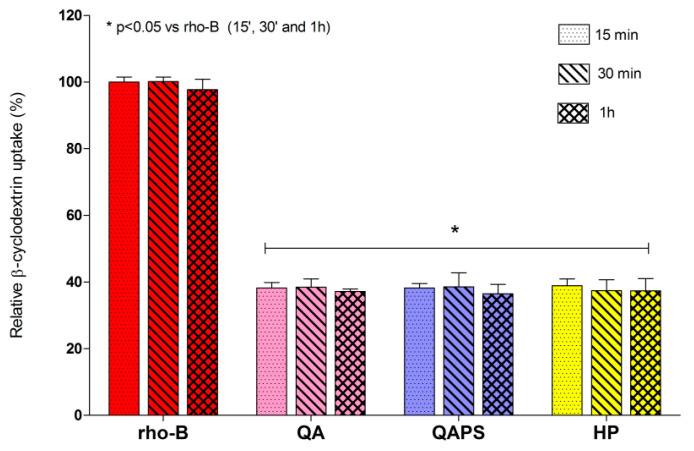
Relative beta-cyclodextrin uptake in CACO-2 cells. Evaluation of the intracellular fluorescence in CACO-2 cells after exposure to RBITC-HP, RBITC-QA and RBITC-QAPS (5 µM) for 15, 30 and 60 min. Data are reported as mean ± SD (*n* = 3). * *p* < 0.05 vs. rho-B 0.5 µM at 15, 30 and 60 min (ANOVA test).

The beta-cyclodextrin absorption was also evaluated using RBITC-HP, RBITC-QA and RBITC-QAPS (5 µM) at 15, 30 and 60 min of exposure in CACO-2 cells seeded in 96-multiwell plates (Figure 4). The fluorescence intensity recorded in the cell lysates did not show any significant difference between the samples at increasing time. In fact, the uptake expressed as relative beta-cyclodextrin uptake percentage was around 38% for all samples.

### 3.4. Uptake and Transepithelial Permeability Evaluation in Cell Monolayers

**Figure 5:**
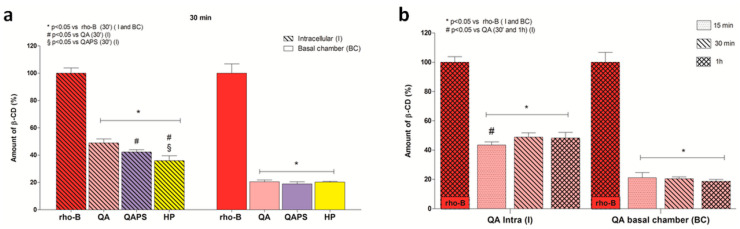
Evaluation of transepithelial permeability of RBITC-HP, RBITC-QA and RBITC-QAPS (5 µM) in CACO-2 cell monolayers. (**a**) Amount of cyclodextrins accumulated into the cells and in the basolateral compartment at 30 min. * *p* < 0.05 vs rho-B - I and BC; # *p* < 0.05 vs. RBITC-QA - I; § *p* < 0.05 vs. RBITC-QAPS - I (ANOVA test). (**b**) Amount of RBITC-QA accumulated into the cells and in the basolateral compartment at 15, 30 and 60 min. * *p* < 0.05 vs. rho-B - I and BC; # *p* < 0.05 vs. 30 min - I and 60 min - I (ANOVA test). Data are reported as mean ± SD (*n* = 3).

To study the permeability in CACO-2 cell monolayers, the fluorescent RBITC-HP, RBITC-QA and RBITC-QAPS (5 µM) were used at 30 min of exposure (Figure 5a). During the experiments, no changes in monolayer integrity were recorded according to TEER values. The amount of beta-cyclodextrins (%) was directly proportional to the fluorescence intensity. Figure 5a shows that the intracellular fluorescence intensity (I) at 30 min follows this order—RBITC-QA (49%) > RBITC-QAPS (42%) > RBITC-HP (36%). Moreover, in the basolateral chamber (BC), no significant difference was identified between the fluorescence intensity of the different cyclodextrins. On the basis of this result, the permeability of RBITC-QA in CACO-2 cell monolayers was studied also at 15 and 60 min of exposure. As shown in Figure 5b, RBITC-QA was able to quickly cross the cell membrane and accumulate into the cells within 15 min, after which the intracellular amount of RBITC-QA increased and reached the maximum at 30 min.

**Figure 6:**
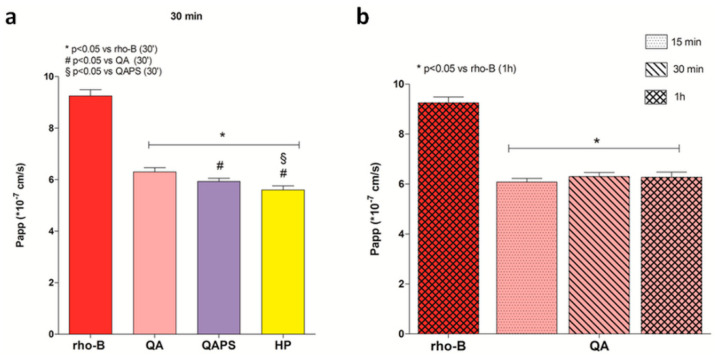
Evaluation of transepithelial permeability of RBITC-HP, RBITC-QA and RBITC-QAPS in CACO-2 cell monolayers. (**a**) The apparent permeability (P_app_) values at 30 min. * *p* < 0.05 vs. rho-B; # *p* < 0.05 vs. RBITC-QA; § *p* < 0.05 vs. RBITC-QAPS (ANOVA test). (**b**) The apparent permeability (P_app_) of RBITC-QA at 15, 30 and 60 min. * *p* < 0.05 vs rho-B (ANOVA test). Data are reported as mean ± SD (*n* = 3).

The Figure 6 shows the transepithelial permeability values of the studied beta-cyclodextrins. The obtained data showed that transepithelial permeability of RBITC-QA at 30 min is higher than that measured for RBITC-HP and RBITC-QAPS.

### 3.5. Studies of Protective Effects

**Figure 7:**
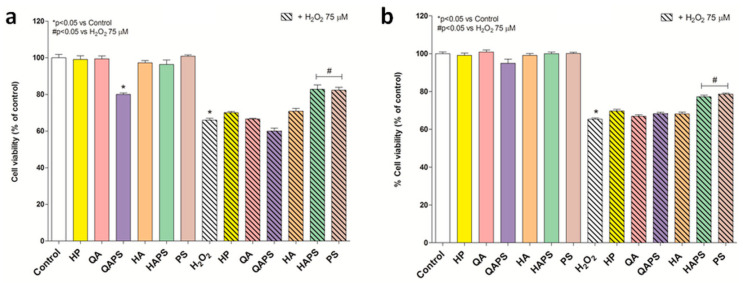
Effects of different beta-cyclodextrins in PC12 and CACO-2 cells exposed to hydrogen peroxide. MTT analysis was performed on PC12 (**a**) and CACO-2 cells (**b**) after 24 h exposure to HP, QA, QAPS, HA, HAPS and PS (0.5 µM) alone or with H_2_O_2_ 75 µM. * *p* < 0.05 vs. Control; # *p* < 0.05 vs H_2_O_2_ 75 µM (ANOVA test). Data are reported as mean ± SD (*n* = 3).

As shown in Figure 7, the protective effect of HP, QA, QAPS, HA, HAPS and PS (0.5 µM) in PC12 and CACO-2 cells exposed to hydrogen peroxide (75 µM) for 24 h was evaluated and compared. In the presence of HAPS and PS, the H_2_O_2_-induced damage was reduced to 18% and 17% in PC12 cells, respectively, and 12% and 14% in CACO-2 cells, respectively.

## 4. Discussion

The nasal cavity is a versatile site for drug administration because systemic and/or brain targeting can be achieved depending on the deposition site. From the respiratory epithelium, drugs can reach blood circulation or the endings of the trigeminal nerve and thus the brain; from the olfactory epithelium a drug can have a direct access to the cerebrospinal fluid (CSF) or the brain through the olfactory neurons or the olfactory epithelial cells [23]. Taking into account the structure of this epithelium, cytotoxicity studies were carried out using PC12 and CACO-2 cells as models for the neuronal and epithelial cells, respectively. Our primary objective, in fact, was to demonstrate the biocompatibility of cationic beta-cyclodextrin monomers and polymers and thus suggesting their use for nasal formulations. All cationic beta-cyclodextrin polymers, QAPS and HAPS, show dose- and time-dependent toxicity; nevertheless, at 5 µM concentration and 60 min of exposure, the cell viability value is about 80% for QAPS and thus it could be recognized as nontoxic [24,25,26]. HAPS has low toxic effect at 5 µM concentration and 60 min of exposition. The exposure time of 60 min is considered enough for nasal excipients/drugs due to the fast nasal mucociliary clearance; mucociliary transit time in humans is normally 12 to 15 min [27]. As expected, the in vitro cytotoxicity of cationic beta-cyclodextrin polymer was higher than PS and respective monomers (HA and QA) and HP. These results are related to the charge and density of charge of the macromolecules and their molecular weight. As reported by Fischer and co-workers, the molecular weight as well as the cationic charge density affect the interaction with the cell membranes and thus, the cell damage [28]. The positive charge of the macromolecules enables the electrostatic interactions with the negatively charged components of the cell membrane, which disturbs the membrane structure and function, and, consequently, the metabolic activity of cells [28,29]. It follows that the higher number of charges of the macromolecules determine a greater degree of interaction and, thus, a higher damaging effect (HA versus QA; HAPS versus QAPS). The molecular weight is correlated with the toxicity of polymers in respect to the monomers (QAPS versus QA; HAPS versus HA). Moreover, the permanently charged macromolecules, containing the quaternary ammonium groups (QA and QAPS), exhibit a lower toxicity than those with protonable primary and secondary amines (HA and HAPS) [28,30].

Cell viability has been tested also after 24 h incubation time in order to predict the long-term toxicity. The toxicity of HAPS is unchanged after 24 h treatment time, whereas that of QAPS increases and is pronounced in PC12 compared with CACO-2 (*p* < 0.05). This behaviour is probably related to the molecular weight. In fact, the cytotoxicity of cationic chains with a medium range of molecular weight (1.7–3.9 × 10^4^ g/mol) is related to the destabilization of the cellular membrane, whereas cationic polymers with long chains (Mw > 3.9 × 10^4^ g/mol) are more toxic in the intracellular space, but longer time and repeated exposure are required [31].

Based on in vitro cytotoxicity, QAPS and QA were selected for uptake studies. Fluorescently labelled HP (RBITC-HP) was tested as comparison. The endocytosis of fluorescently labelled random methyl-beta-cyclodextrin, (2-hydroxypropyl)-beta-cyclodextrin and soluble beta-cyclodextrin polymer cross-linked with epichlorohydrin has been already revealed [32,33]. CACO-2 cells were chosen because they grow in culture as an adherent monolayer of epithelial cells compared to PC12 and they are suitable for transport studies [34]. The uptake evaluated by confocal microscopy showed that after 30 min, all monomeric and polymeric fluorescent beta-cyclodextrins were internalized in the cytoplasm of CACO-2 cells with different efficiency. In fact, the RBITC-QA crossed biological membranes better than the polymer RBITC-QAPS. These results were confirmed and quantified by the experiments of transepithelial permeability on CACO-2 cell monolayers. In particular, RBITC-QA showed higher transepithelial permeability than RBITC-HP and the polymer RBITC-QAPS. Whereas, the uptake measured in 96-multiwell plates did not show differences between beta-cyclodextrins at different concentrations and times of exposure; this effect was probably due to a limit in the sensitivity of the method. The obtained data confirmed a good availability of QA and QAPS to cross biological membranes and a valid uptake of these in the cell environment. It means that also QAPS as an excipient can improve the nasal absorption of drugs by promoting the transport into the cells within 15 min. This is an important property for nasal drug delivery systems to overcome the poor permeability of nasal mucosa and the short absorption time available due to fast mucociliary clearance. The contact time of drugs with the mucosa could be also increased by the electrostatic interactions between the cationic beta-cyclodextrin and sialic acid resides of mucin.

The results obtained warrant further research on a possible intrinsic pharmacological action of these cationic beta-cyclodextrins and to study their possible role as adjuvants in pharmacological therapies or as active pharmaceutical ingredients. In this regard, the activity of the molecules on oxidative stress induced by H_2_O_2_ was evaluated in both cell lines, PC12 and CACO-2—the protective effect of the two polymers HAPS and PS was shown. The HAPS and PS reduced oxidative damage in PC12 and CACO-2 cells. This new property is particularly interesting and gives a potential antioxidant activity to HAPS and PS, which could be used in the prevention and/or treatment of oxidative stress-related neurodegenerative diseases. If administered nasally, these cationic polymers could directly reach the brain by nose to brain transport and perform this pharmacological activity. Nevertheless, the mechanism that causes this antioxidant effect is unknown and therefore it is the subject of further studies currently in progress.

## 5. Conclusions

The cytotoxicity and cell uptake studies indicated that cationic beta-cyclodextrins monomers and polymers possess low toxicity, a good uptake and a different permeability index depending on the density of charge and the molecular weight. In particular, QA and QAPS could be regarded as useful nasal excipients able to improve the absorption of drugs. Moreover, the preliminary studies suggest a potential protective effect against oxidative stress of HAPS and PS in both cell lines, supposing an intrinsic action which needs to be deeply investigated.

## Figures and Tables

**Table 1:** Description of beta-cyclodextrin monomers and polymers studied in this work.

Cyclodextrin	Code	DS ^1^	MW ^2^	CLR ^3^	CD ^4^	Analogue Fluorescent Cyclodextrin
(2-Hydroxy-3-N,N,N-trimethyl amino) propyl-beta-cyclodextrin chloride	QA	3	1589.8		3	RBITC-QA
Quaternary-ammonium-beta-cyclodextrin soluble polymer crosslinked with epichlorohydrin	QAPS	2.2	40,000	~11	2.2	RBITC-QAPS
Heptakis (6-deoxy-6-amino)-beta-cyclodextrin heptahydrochloride	HA	-	1383.3		7	
Soluble amino-beta-cyclodextrin polymer crosslinked with epichlorohydrin	HAPS	1	25,000	~10	1	
(2-Hydroxypropyl)-beta-cyclodextrin	HP	4.5	1400			RBITC-HP
Soluble β-cyclodextrin polymer crosslinked with epichlorohydrin	PS	-	92,000	~11		RBITC-PS

^1^ DS: Average Degree of Substitution; ^2^ MW: Average Molecular Weight (g/mol); ^3^ CLR: Cross-Linking Ratio (mol epichlorohydrin /mol CD); ^4^ CD: Cationic Density (cationic groups per cyclodextrin unit).

## Supplementary Materials

The following are available online at https://www.mdpi.com/1999-4923/12/7/658/s1, Table S1: Description of beta-cyclodextrin monomers and polymers studied in this work. Figure S1. Cartoon representations for beta-cyclodextrin monomers: (a) HP; (b) HA; (c) QA; (d) RBITC-HP; (e) RBITC-QA, Figure S2: Cartoon representations for beta-cyclodextrin polymers: (a) PS; (b) HAPS; (c) QAPS; (d) RBITC-PS; (e) RBITC-QAPS, Figure S3: Effect of different concentrations (0.5–1–2.5–5–10 µM) of HP, QA and QAPS on PC12 cell viability at increasing times of exposure (15, 30, 60 min and 24h). * *p* < 0.05 vs. Control, Figure S4: Effect of different concentrations (0.5–1–2.5–5–10 µM) of HA, HAPS and PS on PC12 cell viability at increasing times of exposure (15, 30, 60 min and 24 h). * *p* < 0.05 vs. Control, Figure S5: Effect of different concentrations (0.5–1–2.5–5–10 µM) of HP, QA and QAPS on CACO-2 cell viability cells at increasing times of exposure (15, 30, 60 min and 24 h). * *p* < 0.05 vs. Control, Figure S6: Effect of different concentrations (0.5–1–2.5–5–10 µM) of HA, HAPS and PS on CACO-2 cell viability at increasing times of exposure (15, 30, 60 min and 24 h). * *p* < 0.05 vs. Control.
