# Supplementary material for: Investigation of Cytotoxicity and Cell Uptake of Cationic Beta-Cyclodextrins as Valid Tools in Nasal Delivery
Source: Pharmaceutics. 2020 Jul 12;12(7):658. doi: 10.3390/pharmaceutics12070658 (PMC7407921; doi:10.3390/pharmaceutics12070658)
Supplement: Supplementary file 1 [file pharmaceutics-12-00658-s001.pdf]

pharmaceutics

Supplementary Materials: Investigation of Cytotoxicity and Cell Uptake of Cationic Beta-Cyclodextrins as Valid Tools in Nasal Delivery

Giovanna Rassu, Silvia Fancello, Marta Roldo, Milo Malanga, Lajos Szente, Rossana Migheli *, Elisabetta Gavini * and Paolo Giunchedi

Table S1. Description of beta-cyclodextrin monomers and polymers studied in this work.

Not Fluorescent							Analogue fluorescent cyclodextrin					
Cyclodextrin	Code	DS[1]	MW[2]	CLR[3]	CD[4]	Description	Cyclodextrin	Code	DS[1]	MW[2]	CLR[3]	CD[4]
(2-Hydroxy-3-N,N,N-trimethylamino) propyl-beta- cyclodextrin chloride	QA	3	1589.8	-	3	cationic monomer; purity > 99%	Quaternary ammonium-6-deoxy- 6-[(5/6)-rhodaminyl thioureido]-(2-Hydroxy-3-N,N,N-trimethylamino)-beta-cyclodextrin	**RBITC-QA**	4.5 (QA) 1 (RBITC)	1939.86	-	3.5
Quaternary-ammonium-beta- cyclodextrin soluble polymer crosslinked with epichlorohydrin	QAPS	2.2	40,000	~11	2.2	cationic polymer; CD content: 50-70%	Quaternary-ammonium-rhodamine labeled beta-cyclodextrin soluble polymer crosslinked with epichlorohydrin	**RBITC-QAPS**	2.2 (QA) 0.05 (RBITC)	40,000	~11	2.2
Heptakis (6-deoxy-6-amino)-beta-cyclodextrin heptahydrochloride	HA	-	1383.3	-	7	cationic monomer; purity > 98%						
Soluble amino-beta-cyclodextrin polymer crosslinked with epichlorohydrin	HAPS	1	25,000	~10	1	cationic polymer; CD content: 70%						
(2-Hydroxypropyl)- beta-cyclodextrin	HP	4.5	1400	-	-	control monomer	6-deoxy-6-[(5/6)-rhodaminylthioureido]-hydroxypropyl-beta-cyclodextrin	**RBITC-HP**	4.7 (HP) 0.5 (RBITC)	1675.07		1*
Soluble β-cyclodextrin polymer crosslinked with epichlorohydrin	PS	-	92,000	~11	-	control polymer; CD content: 70%	Rhodamine labeled BCD soluble polymer crosslinked with epichlorohydrin	**RBITC-PS**	0.05 (RBITC)	100,000	~11	-

[1] DS: Average Degree of Substitution; [2] MW: Average Molecular Weight (g/mol); [3] CLR: Cross-Linking Ratio (mol epichlorohydrin/mol CD); [4] CD: Cationic Density (cationic groups per cyclodextrin unit). * anionic group per cyclodextrin unit

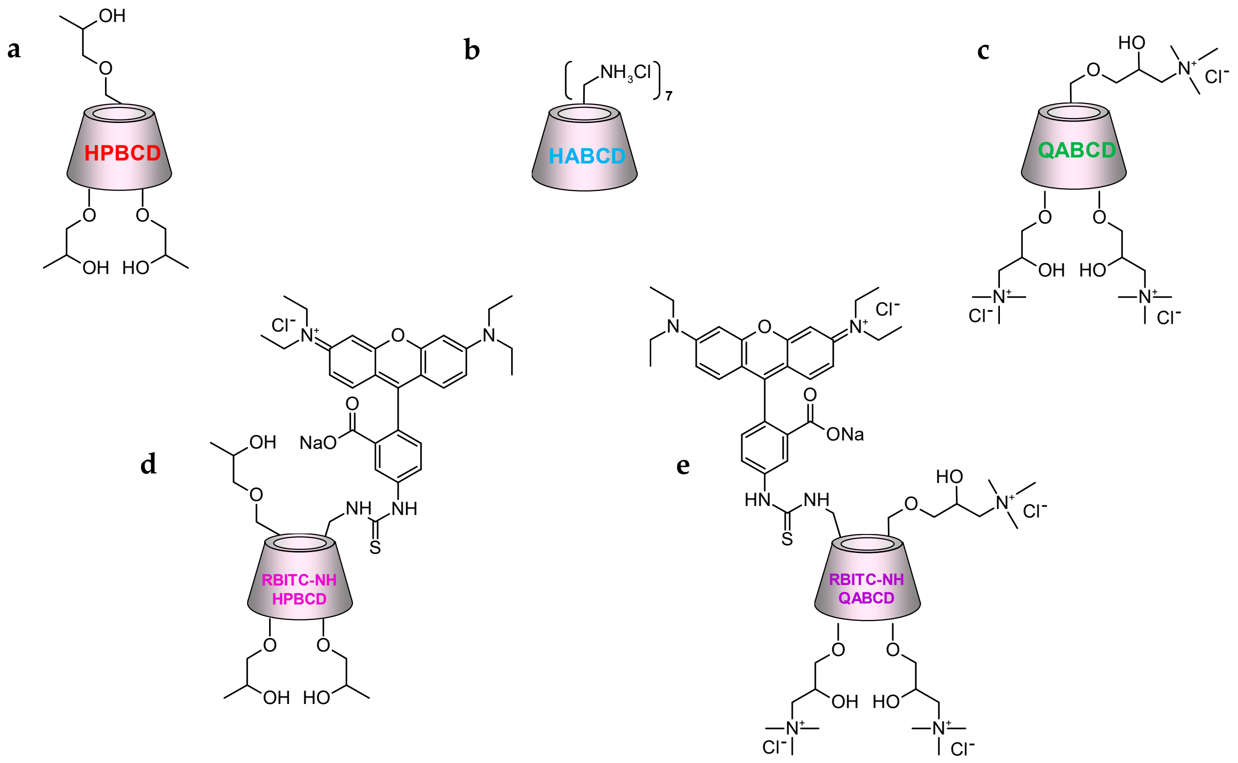

Figure S1. Cartoon representations for beta-cyclodextrin monomers: (**a**) HP; (**b**) HA; (**c**) QA; (**d**) RBITC-HP; (**e**) RBITC-QA.

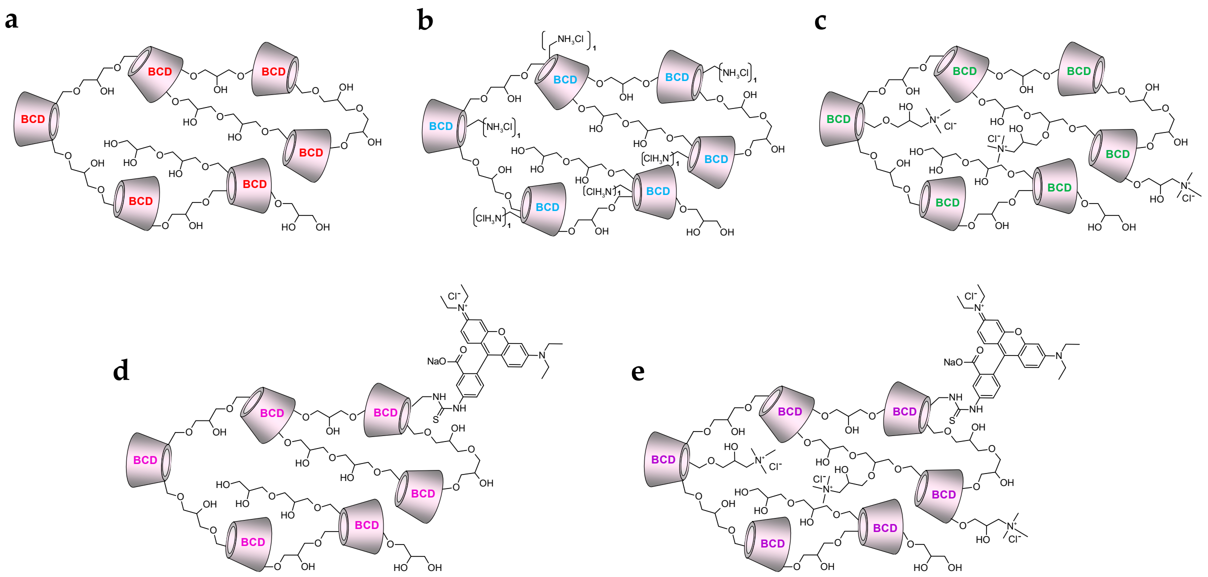

Figure S2. Cartoon representations for beta-cyclodextrin polymers: (**a**) PS; (**b**) HAPS; (**c**) QAPS; (**d**) RBITC-PS; (**e**) RBITC-QAPS.

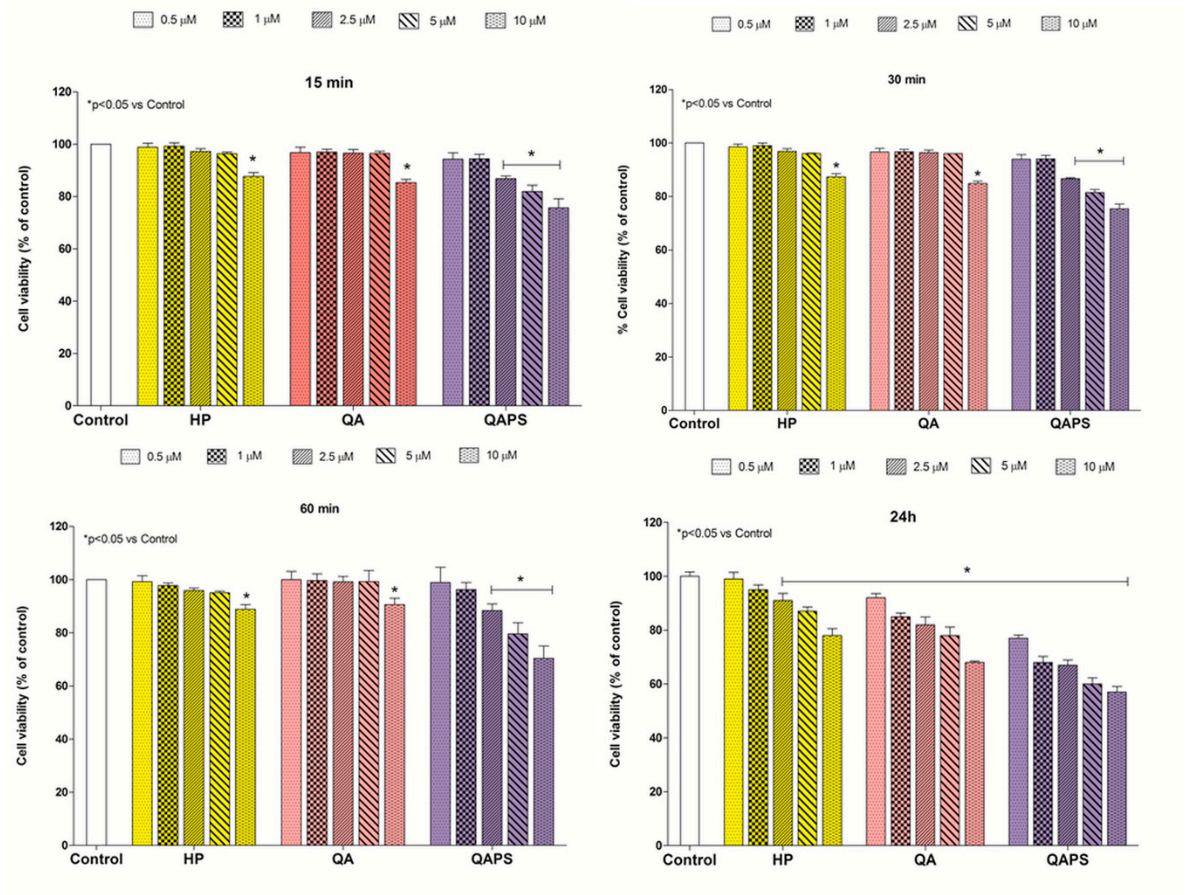

Figure S3. Effect of different concentrations (0.5 - 1 – 2.5 - 5 - 10 µM) of HP, QA and QAPS on PC12 cell viability at increasing times of exposure (15, 30, 60 min and 24h). *$p < 0.05$ vs Control.

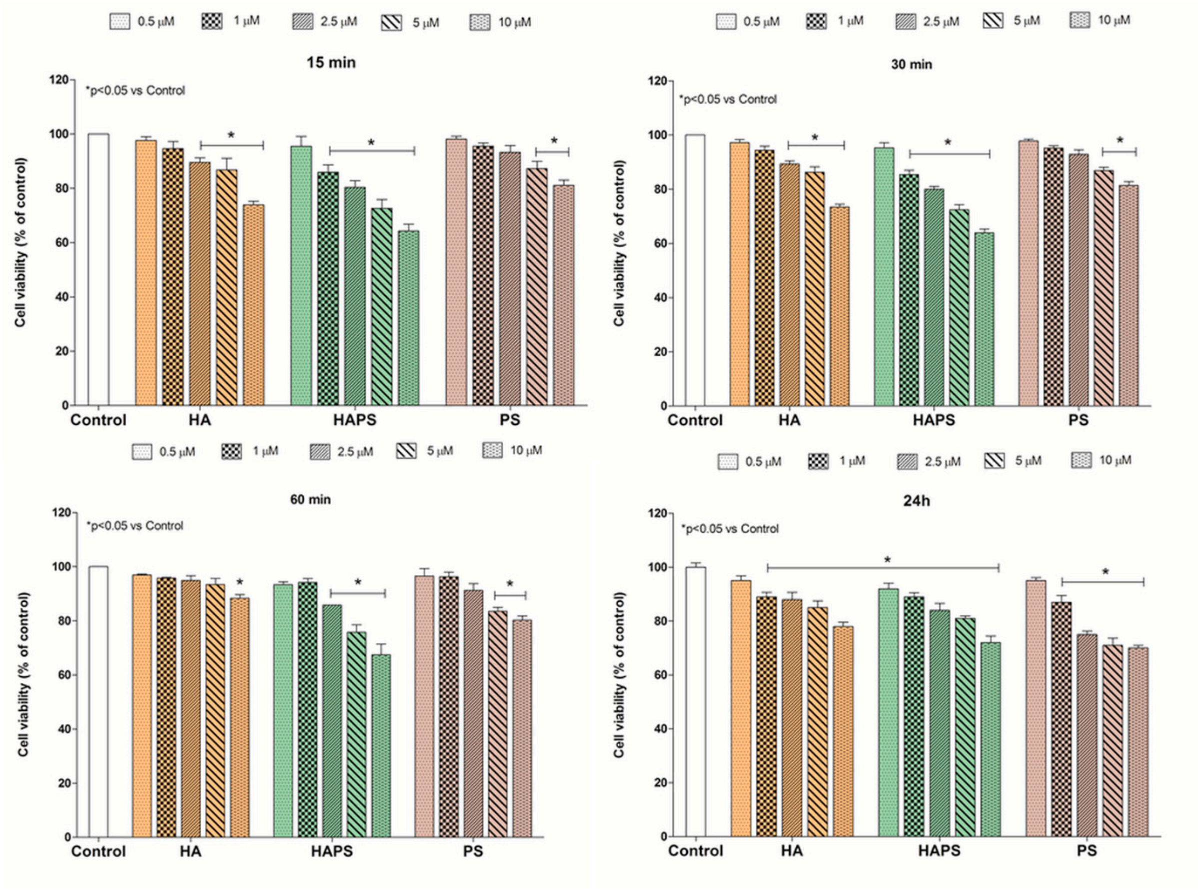

Figure S4. Effect of different concentrations (0.5 - 1 – 2.5 - 5 - 10 μM) of HA, HAPS and PS on PC12 cell viability at increasing times of exposure (15, 30, 60 min and 24h). $*p < 0.05$ vs Control.

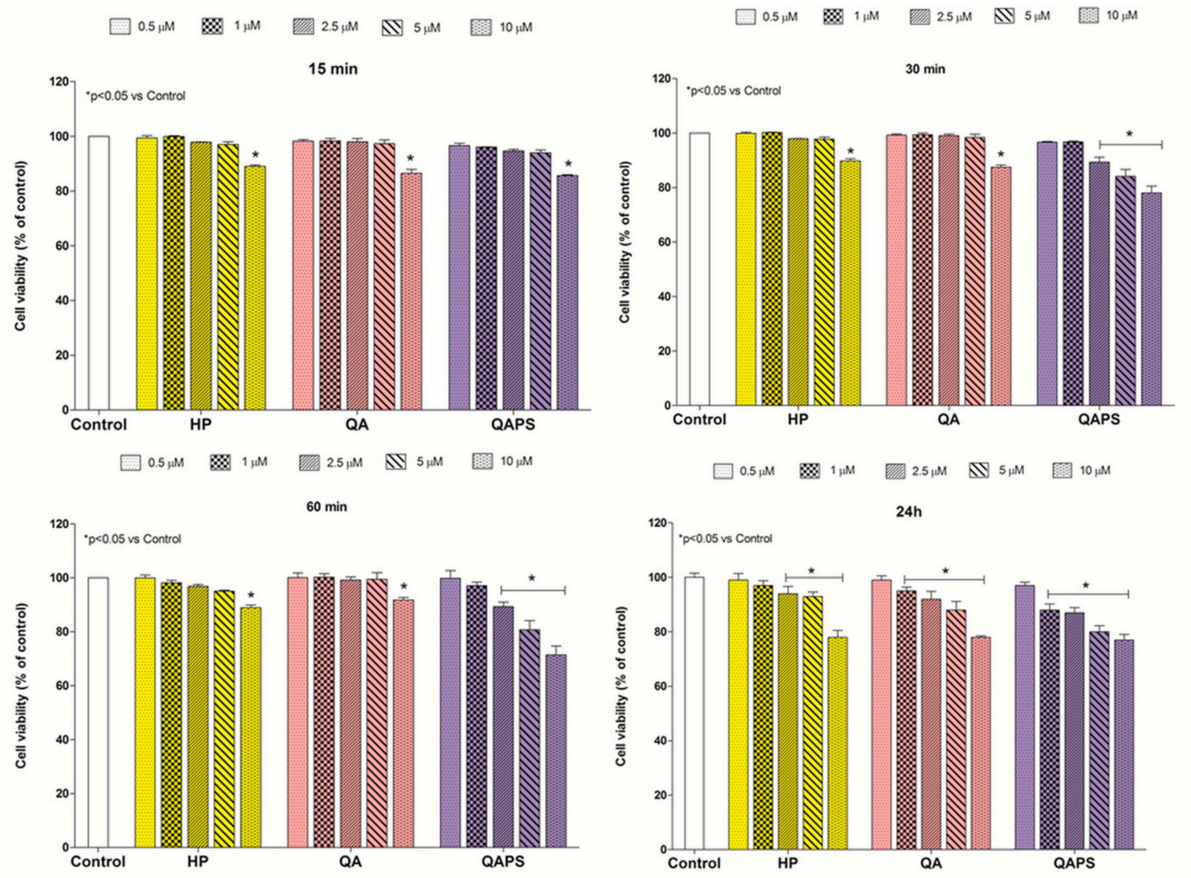

Figure S5. Effect of different concentrations (0.5 - 1 – 2.5 - 5 - 10 μM) of HP, QA and QAPS on CACO-2 cell viability at increasing times of exposure (15, 30, 60 min and 24h). *$p < 0.05$ vs Control.

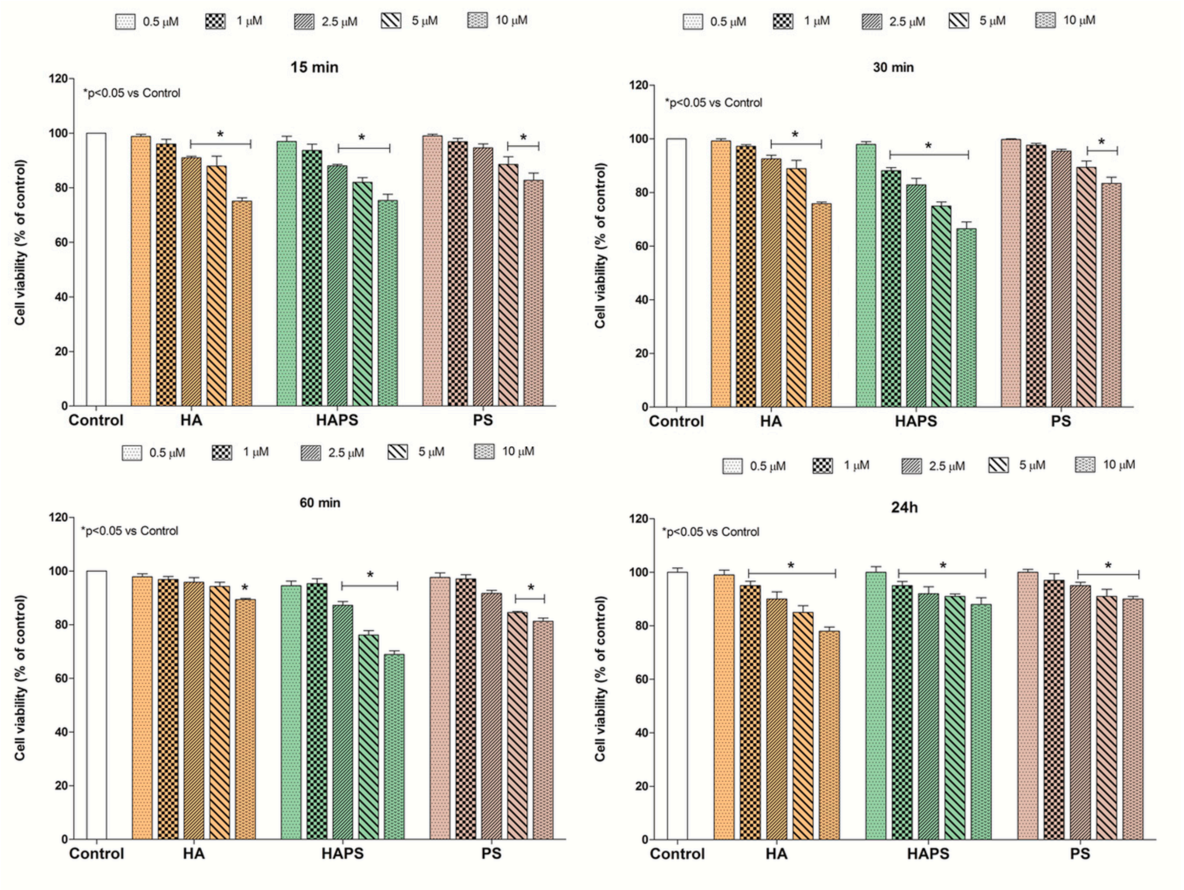

Figure S6. Effect of different concentrations (0.5 - 1 – 2.5 - 5 - 10 μM) of HA, HAPS and PS on CACO-2 cell viability at increasing times of exposure (15, 30, 60 min and 24h). *$p < 0.05$ vs Control.
